# Central Retinal Artery Occlusion Associated with Carotid Artery Occlusion

**DOI:** 10.5811/cpcem.2019.4.40847

**Published:** 2019-05-21

**Authors:** Wells Weymouth, Craig Pedersen

**Affiliations:** San Antonio Military Medical Center, Department of Emergency Medicine, Fort Sam Houston, Texas

## Abstract

Sudden, painless vision loss in patients with stroke risk factors is suspect for central retinal artery occlusion (CRAO), an ophthalmic emergency that in addition to ocular treatment warrants a thorough neurologic and vascular evaluation. In addition to the high risk of concurrent stroke, carotid artery stenosis and occlusion is often overlooked during the initial evaluation. Here we report a case of CRAO with concurrent ipsilateral complete left internal carotid artery (ICA) occlusion and right ICA critical narrowing, dissection and pseudoaneurysm, which subsequently improved with prompt hyperbaric oxygen therapy.

## INTRODUCTION

Known commonly as a “stroke of the eye,” central retinal artery occlusion (CRAO) presents with sudden, severe, painless monocular visual loss, and is an ophthalmic emergency. While the presentation is rare (the incidence of CRAO is estimated around 1.9/100,000 in the United States) the consequences can be dire.^1^ The strong association between CRAO and stroke is well known, and prompt evaluation of the carotid arteries is paramount in order to expedite definitive treatment, especially since ipsilateral carotid artery disease is known to occur in 50% of these patients.^2^,^3^

## CASE REPORT

A 66-year-old male with known history of hypertension and hypothyroidism presented to the emergency department (ED) with sudden, painless loss of vision in his left eye of two hours duration. The patient reported that he had sneezed four times in rapid succession and then stated he lost vision in his left eye, but experienced no eye pain. The patient then drove himself to the ED. He denied any other accompanying symptoms on extensive review of systems. Examination revealed 20/30 vision in his right eye with corrective lenses (eyeglasses), but no light perception in his left. The left pupil did not have any direct light response but had preserved consensual response. Consensual light response was absent in the right pupil, but direct response was preserved. Point-of-care ocular ultrasound was performed but did not reveal any apparent abnormality. The patient had no other focal neurological deficits and was found to be in normal sinus rhythm on electrocardiogram. Tonometry was not available at this facility nor were ophthalmologic services. A head computed tomography (CT) without contrast showed no acute hemorrhage or apparent infarct and he was sent emergently to the ophthalmology clinic at the tertiary care hospital several miles away in consultation with the receiving ophthalmologist.

In the ophthalmology clinic, the patient was confirmed to have multiple arterial thrombi with characteristic cherry-red spot on exam consistent with CRAO. He was then transferred directly from the ophthalmology clinic to the hyperbaric chamber for hyperbaric oxygen therapy. Upon the initial dive at 33 feet of therapy he began seeing letters and his vision continued to improve after one hour at 60 feet. Vision in the affected eye improved from solely light perception to 20/50 at 24 hours, with subsequent resolution of symptoms. Shortly thereafter, while inpatient he received CT angiography of the neck and head (Image), which revealed complete left internal carotid artery (ICA) occlusion at its origin with retrograde filling of the distal cervical and intracranial portions. The right ICA showed regions concerning for dissection with associated pseudoaneurysm formation, with another region just distal with critical narrowing of the cervical internal carotid artery to approximately one millimeter.

Several days later the patient underwent successful transcarotid artery revascularization in the operating room.

## DISCUSSION

In the setting of sudden, painless vision loss, the diagnosis of CRAO must be at the top of the differential, as it portends the most vision- and life-threatening underlying diagnosis and with associated high mortality. Typical patients have severe monocular visual loss, with 80% of patients having a visual acuity of 20/400 or worse as a result of loss of blood supply to the inner retinal layers.^4^ Analogous to ischemic cerebral stroke, the pathology is thromboembolic and the majority are due to carotid artery disease, primarily due to atherosclerotic plaques. As demonstrated in this case, one cannot neglect carotid stenosis (and the heart) as other potential sources of emboli.

Risk factors mimic those of cerebral stroke and include hypertension, diabetes mellitus, carotid artery disease, coronary artery disease, a history of transient ischemic attacks or cerebral vascular accidents, and smoking tobacco, among others.^5^ In fact, atherosclerotic disease is the leading cause of CRAO in patients aged 40–60 years.^6^ There have also been several case series of CRAO during and post angiography and stenting.^7^,^8^ Patients typically present with crippling monocular vision loss, and clinicians should focus on detailed ophthalmic and neurologic exams. As in this case, the classic “cattle trucking” or “box-carring,” which is a discontinuous appearance of the vessels due to segmentation of the blood column in the arteries, and the cherry-red spot in the macula, may be difficult to see without a dilated fundoscopic exam.^8^

Given the emergent nature of the condition, swift imaging and treatment is critical. It is suspected that irreversible retinal damage occurs without recovery of vision within six and a half hours, although animal models show partial recovery only up to 240 minutes.^9^,^10^ Given these constraints, it is critical to obtain an expeditious CT to assess for intracranial infarct as soon as possible. In at least one study, 32% had acute or subacute brain infarct seen on imaging.^11^ Given the propensity for arterial occlusion and severe carotid stenosis (up to 40% of patients), we would recommend CT angiography of the neck and head be done as well, which is consistent with current guidelines.^12^ One study demonstrated that nine of nine patients with CRAO had angiographically demonstrable ipsilateral carotid artery disease.^13^ As magnetic resonance imaging is more time consuming, it was this team’s opinion that it could be done while inpatient.

**CPC-EM Capsule**

What do we already know about this clinical entity?

Central retinal artery occlusion is an ophthalmic emergency which requires both prompt neurologic and ophthalmic evaluation and advanced imaging.

What makes this presentation of disease reportable?

Data on treatment efficacy for tissue plasminogen activator, hyperbaric treatment, and anterior chamber paracentesis is limited.

What is the major learning point?

Hyperbaric oxygen therapy is an underutilized treatment, and if available, should be strongly considered within eight hours.

How might this improve emergency medicine practice?

After central retinal artery occlusion is recognized, perform advanced imaging, and in conjunction with ophthalmic consultation, consider hyperbaric oxygen.

Treatment for CRAO targets acute reperfusion of the central retinal artery; however, the exact choice of treatment is still a matter of debate. In this case, the physicians chose hyperbaric treatment and achieved vision recovery. The use of this modality is relatively rare; it is offered only 7% of the time, even though based on the American Heart Association classification of evidence, treatment of CRAO with hyperbaric oxygen therapy is Level IIb.^14^,^15^ Literature review demonstrates that overall 65% of cases have shown improvement when treated with hyperbaric oxygen, although it is apparent that a significant portion of patients will benefit from oxygen alone. Given the relative ubiquity of supplemental oxygen and relatively low risk, it seems logical to place the patient on 100% oxygen by nasal cannula or non-rebreather upon presentation. However, if there is no significant improvement with normobaric oxygen within 15 minutes, one algorithm proposes to proceed immediately to hyperbaric oxygen at two atmospheres of pressure.^16^

As with most instances of ischemia, the colloquialism “time is vision” may apply here as well. Best outcomes likely result when applied within the first eight hours from the onset of visual impairment.^17^ The most widely studied treatment is tissue plasminogen activator, which binds to the site of the thrombus and facilitates the conversion of plasminogen to plasmin to dissolve the clot. Given the similarities between ischemic stroke and CRAO, it does seem biologically plausible it would be effective. It would appear, however, that data on the subject are mixed, with at least two large reviews demonstrating improvement of visual acuity as well as relative safety, and two other large multicenter randomized controlled trials failing to show the same but demonstrating severe side effects including the feared complication of intracranial hemorrhage.^9^,^18^–^22^ Another treatment that is well described and commonly used although its efficacy is unproven, is anterior chamber paracentesis. It functions to lower intraocular pressure to increase dilatation of the retinal arteries due to vascular tortuosity produced from distortion of the globe.^23^ Unfortunately, a 2009 Cochrane review found no treatment modality is more effective than placebo.^24^

Even after treatment, if any, these patients remain at high risk for ischemic stroke, with the incident rate ratio peaking at one to seven days after CRAO and remaining elevated for the first 30 days.^25^

## CONCLUSION

Prompt recognition of CRAO symptoms should be followed by a detailed neurologic and vascular evaluation for concurrent stroke and carotid artery stenosis or occlusion. These patients are also at risk for ischemic events after treatment, largely because atherosclerotic disease is the primary underlying diagnosis for both conditions. Hyperbaric oxygen is an underused treatment modality with promising results. Patients presenting to the ED within eight hours of symptom onset should be considered for this therapy. This case highlights the high risk to these patients, which requires intensive workup and admission from the ED and while inpatient, as well as provides further evidence for the successful use of hyperbaric oxygen treatment.

## Figures and Tables

**Image:**
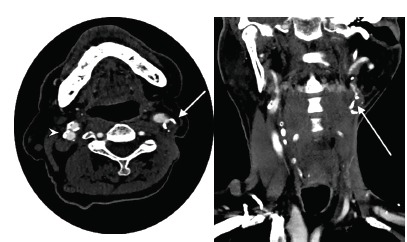
Computed tomography angiography of the head and neck in coronal (right) and axial (left) views demonstrating complete occlusion of the left internal carotid artery (ICA) (arrows) and critical narrowing of the right ICA (arrowhead), in a patient with right central retinal artery occlusion.
